# Rare association between giant-cell aortitis and giant-cell aortic valvulitis

**DOI:** 10.4322/acr.2023.449

**Published:** 2023-10-23

**Authors:** Georgina del Cisne Jadán Luzuriaga, Ricardo Ribeiro Dias, José Augusto Duncan Santiago, Vagner Madrini, Walther Yoshiharu Ishikawa, Fabio Fernandes, Vera Demarchi Aiello

**Affiliations:** 1 Universidade de São Paulo, Faculdade de Medicina, Instituto do Coração, Departamento de Miocardiopatias e Doenças da Aorta, São Paulo, SP, Brasil; 2 Universidade de São Paulo, Faculdade de Medicina, Instituto do Coração, Departamento de Cirurgia Cardiovascular, São Paulo, SP, Brasil; 3 Universidade de São Paulo, Faculdade de Medicina, Instituto do Coração, Departamento de Imagem Cardiovascular, São Paulo, SP, Brasil; 4 Universidade de São Paulo, Faculdade de Medicina, Instituto do Coração, Departamento de Anatomia Patológica, São Paulo, SP, Brasil

**Keywords:** Aorta, Thoracic, Aortic Diseases, Aortic Aneurysm, Aortic Valve, Aortic Valve Insufficiency

## Abstract

Giant cell arteritis (GCA) is a type of chronic vasculitis that affects medium and large-caliber arteries, frequently related to aortic involvement and, consequently, to aneurysm formation. However, associated valvulitis with giant cells is uncommon. We describe the case of a 50-year-old female patient with aortic aneurysm and valvular insufficiency, whose anatomopathological examination revealed giant-cell aortic valvulitis associated with giant cell aortitis.

## INTRODUCTION

Giant cell arteritis (GCA) is a vasculitis involving medium and large-caliber arteries.^1^ It is a rare disease, affecting women over 50 years of age more often.^2,3^

Approximately 22% of patients with GCA have aortic involvement (aneurysm/dilation).^4^ In this condition, complications are associated with aortic valvar insufficiency secondary to aortic root dilation, aneurysm and/or aortic dissection, leading to heart failure and death in 20% of cases.^5^

We describe a case of a female patient with aortic aneurysm and valvular insufficiency, whose anatomopathological examination revealed giant-cell aortic valvulitis associated with giant-cell aortitis.

## CASE REPORT

A 50-year-old Caucasian female with no personal or family history of pathological conditions presented with dyspnea on moderate and minor efforts, dizziness, and palpitations. On physical examination, there was an aspiration murmur in the aortic focus (2+/6+), with irradiation to the mitral and accessory aortic foci. Blood count, C-reactive protein, blood sedimentation speed, rheumatoid factor, anti-neutrophil cytoplasmic antibody, and renal function were normal. Serologies for hepatitis B, C, HIV, and syphilis were negative.

The electrocardiogram showed premature ventricular complexes. Chest radiography revealed dilation of the thoracic aorta. The echocardiogram revealed a left atrium of 42 mm, a left ventricle of 64 x 42 mm, and an ejection fraction of 62%, with significant aortic regurgitation and dilation of the ascending aorta.

**Figure 1:**
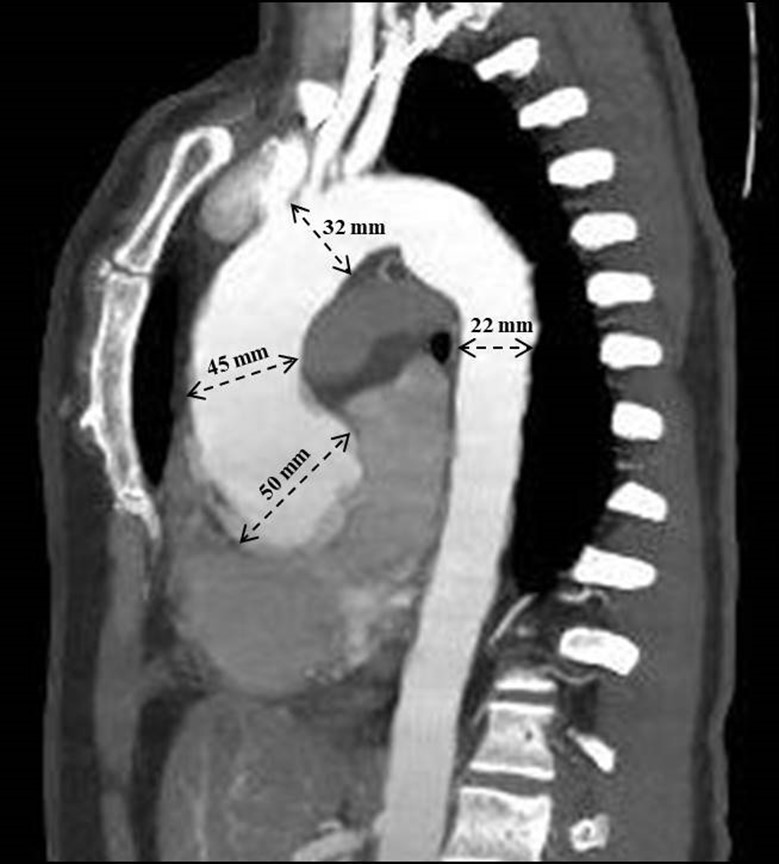
Contrast-enhanced chest CT angiography showing dilatation of the aortic root with a diameter greater than 50 mm in the ascending segment, the ascending segment 45 mm, the aortic arch 32 mm and the descending segment 22 mm.

On angiotomography, the aortic root measured 50 mm (Figure 1), the ascending segment 45 mm, the aortic arch 32 mm, the descending segment 22 mm, and the abdominal segment 17 mm. Surgery for aortic aneurysm correction was indicated. A coronary angiography was requested in the preoperative evaluation, excluding coronary obstructions.

Surgical intervention was performed with aortic root reconstruction and replacement of the ascending aorta (Bentall-De Bono procedure) using a valved tube with a mechanical aortic prosthesis.

**Figure 2:**
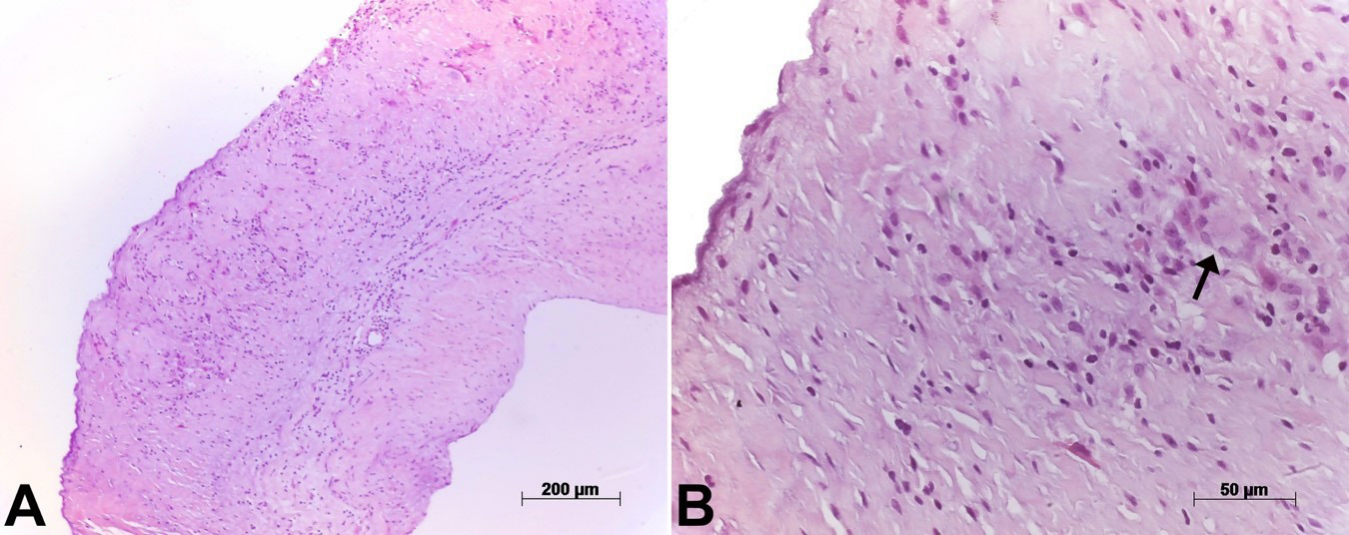
Photomicrographs of an aortic leaflet. In the panel **A -** with diffuse thickening and chronic inflammatory infiltrate; in the panel **B -** the arrows point to a Langerhans-type giant cell within the inflammatory infiltrate. Hematoxylin-eosin staining, objective magnifications 10x and 40x, respectively.

The valve and the aortic aneurysmal segment were sent for anatomopathological analysis. The gross inspection of the aortic valve showed mild fibrous thickening at the free edge of the leaflets. Histological examination of the semilunar leaflets revealed a lymphohistiocytic infiltrate with giant cells, besides focal fibrinoid necrosis. Inflammatory cells were concentrated mainly at the ventricular aspect of the leaflets and spared their free edge. The diagnosis was giant-cell valvulitis with fibrinoid necrosis (Figure 2).

**Figure 3:**
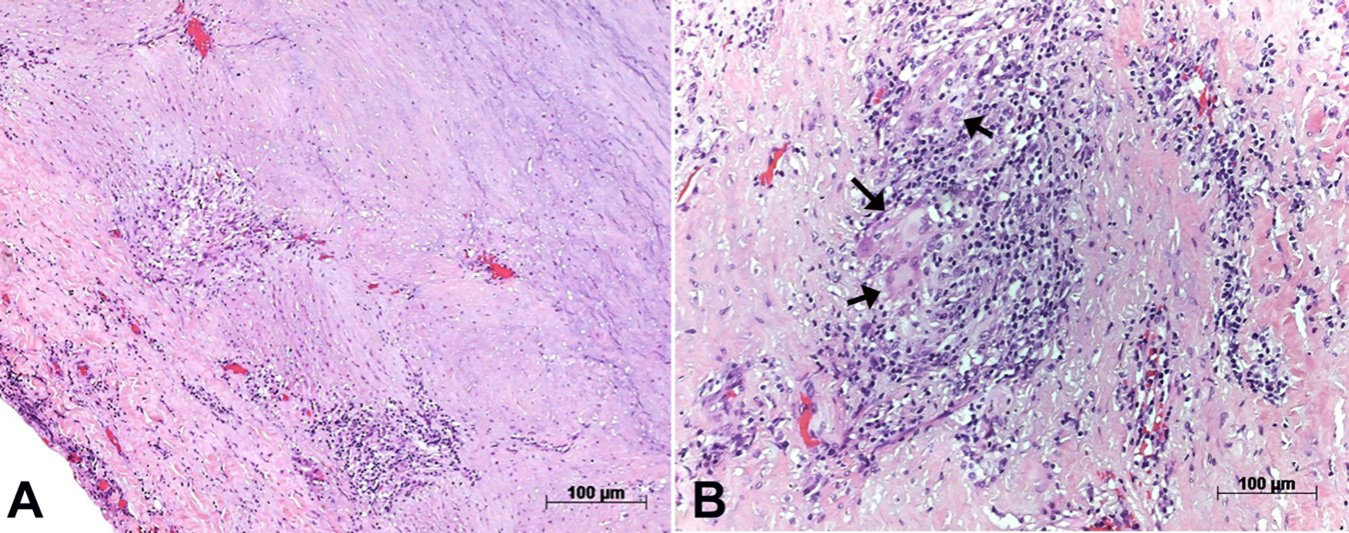
Photomicrographs of the aortic wall showing in panel **A -** the diffuse involvement of the layers by chronic inflammatory infiltrate; in panel **B -** a close-up view shows a giant cell within the inflammatory infiltrate. Hematoxylin-eosin staining, objective magnifications 5x and 40x respectively.

**Figure 4:**
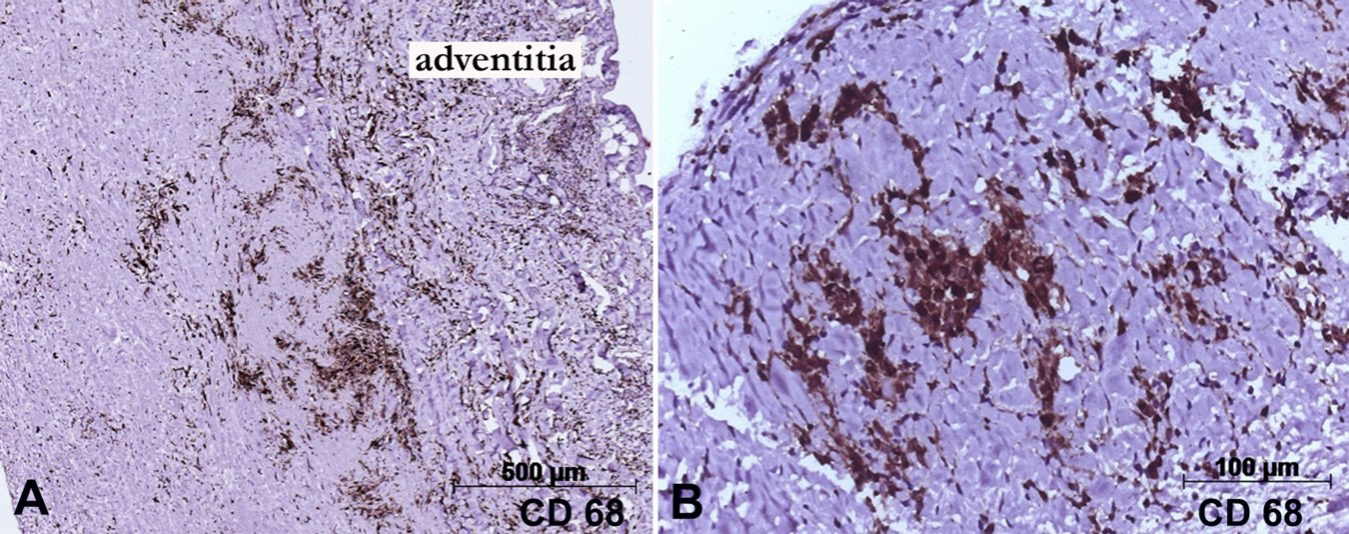
Photomicrographs of the aortic and valvar tissue submitted to immunohistochemical reactions for macrophages (CD68 antigen). In **A -** the positive cells are concentrated in the external half of the aortic wall, near the adventitia; Panel **B -** depicts positive macrophages in one aortic leaflet. Objective magnifications are respectively **A -** 5x; and **B -** 20x. Mouse monoclonal antibody CD68 (clone KP1), counter-staining with Harry’s Hematoxylin.

The aortic wall showed segmental destruction of elastic fibers of the medial layer and diffuse fibrointimal thickening, besides chronic inflammatory infiltrates with Langerhan-type giant cells, which concentrated at the external half of the wall, near the adventitial layer (Figures 3 and 4).

However, well-formed granulomas were not found, in the aortic wall and valvar tissue. Investigations for bacteria, acid-resistant bacilli, and fungi were negative in the valvar leaflets and the aortic wall (Brown-Hopps, Ziehl-Neelsen, and Grocott stains, respectively). CD68 immunostaining was strongly positive in the inflammatory foci in the aorta and valvar leaflets. Postoperative analysis of inflammatory markers showed no significant changes.

## DISCUSSION

The present case describes the uncommon presentation of valvulitis associated with giant cells aortitis observed in the anatomopathological analysis.

When chronic aortitis leads to aortic root dilation, aortic valvar insufficiency usually occurs secondarily.^6^ In a previous review of 386 cases with thoracic aortic aneurysm and aortic insufficiency, only 2,6% were due to GCA, confirmed histopathologically.^7^ Likewise, in another series, from 7551 patients who underwent surgery for ascending or aortic arch disease, only 2% showed GCA on histologic examination.^8^

Involvement of the aortic valve by the same process as the one present in the aortic wall in GCA is sporadic and was first described by Niclauss et al.^9^ and Terré et al.^10^ from Paris also published inflammatory involvement of the aortic valve in GCA, however, in the form of non-infectious endocarditis, with mixed inflammatory infiltrate and absence of giant cells.

It is noteworthy that GCA does not affect the heart valves more frequently, even being a disease of the autoimmune rheumatologic group. However, such impairment may be more significant than reported in the literature since valve replacement is not always performed during GCA surgery.

A prospective study of biopsy or autopsy material in confirmed GCA cases could assess the prevalence of this type of associated inflammatory valvar involvement.

According to the Consensus Statement on Surgical Pathology of the Aorta, this form of aortitis was limited to GCA disease because there were no well-formed granulomas in the anatomopathological analysis, as in other types of arteritis. Likewise, no other clinical manifestations or laboratory or imaging results are suggestive of different forms of vascular disease.^11^

## CONCLUSION

The involvement of the valve and the aortic arterial wall by GCA is an unusual association, and the diagnosis can be determined through the anatomopathological analysis of the specimens. Despite being infrequent, this combination also requires consideration in the differential diagnosis of disorders involving the aorta and aortic valve because its prevalence could be higher in patients who have not yet undergone surgical intervention and that could evolve into aortic syndromes and be life-threatening.
